# Third‐Order Nonlinear Optical Response and Optical Limiting in Silver‐Cobalt Bimetallic Nanoparticles

**DOI:** 10.1002/nap2.70255

**Published:** 2026-08-20

**Authors:** T. K. Nanditha, Shreepooja Bhat, K. B. Manjunatha, Dileep Ramakrishna, K. G. Raghavendra, S. C. Gurumurthy

**Affiliations:** ^1^ Manipal Institute of Technology Manipal Academy of Higher Education Manipal India; ^2^ Department of Physics, NMAM Institute of Technology (NMAMIT) Nitte (Deemed to be University) Nitte India; ^3^ Department of Chemistry RNS Institute of Technology Channasandra Bengaluru India

**Keywords:** bimetallic nanoparticles, excited‐state absorption, nonlinear optics, optical limiting, plasmonic nanomaterials

## Abstract

Developing materials with high third‐order nonlinear susceptibility and low optical limiting thresholds remains a challenge in photonic device engineering. Bimetallic nanoparticles offer a compelling route to enhanced nonlinear optical (NLO) performance by coupling plasmonic field amplification with interfacial electronic structure. Herein, we report the synthesis of AgCo bimetallic nanoparticles with a surface‐confined Co_3_O_4_ layer and a systematic investigation of their third‐order NLO response under both continuous‐wave (CW) and nanosecond pulsed excitation. The system exhibits strong reverse saturable absorption, with β_eff_ reaching 7.5 × 10^−3^ cm/W at 638 nm under CW excitation, *β* = 0.054 m/GW, and a third‐order susceptibility χ^(3)^ = 4.12 × 10^−11^ esu at 532 nm under nanosecond excitation, which is higher than that of many reported molecular and nanostructured systems. Crucially, the intensity‐dependent nonlinear absorption coefficient confirms a sequential excited‐state absorption mechanism rather than two‐photon absorption, driven by hot‐carrier injection across the Ag‐Co_3_O_4_ Schottky junction into mixed‐valence intermediate states. This distinguishes the system from conventional monometallic optical limiters.

## Graphical abstract

Silver‐cobalt bimetallic nanoparticles with a surface Co_3_O_4_ layer combine plasmonic field enhancement with interfacial charge transfer to deliver strong reverse saturable absorption and low‐threshold optical limiting under both continuous‐wave and nanosecond excitation. Intensity‐dependent nonlinear absorption confirms a sequential excited‐state absorption mechanism driven by hot‐carrier injection across the Ag‐Co_3_O_4_ Schottky junction, distinguishing this system from conventional monometallic optical limiters.

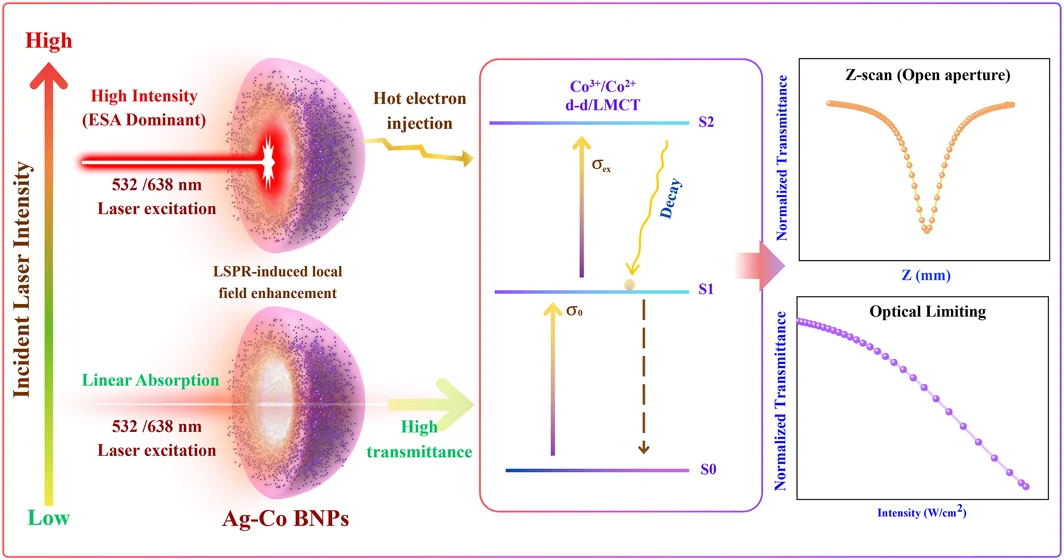

## 1 Introduction

Nonlinear optics (NLO) underpins a wide range of advanced photonic technologies, such as frequency conversion, ultrafast signal processing, and optical limiting. These phenomena arise when the optical response of a material becomes intensity‐dependent under strong electromagnetic fields, leading to nonlinear absorption and refraction. In recent years, the quest for materials with large third‐order nonlinear susceptibilities has intensified owing to their critical role in next‐generation photonic and optoelectronic devices. The rapid development in the fields of nanoscience and nanotechnology has contributed to many advances in this domain [1, 2]. Several nanomaterials have proven to have excellent nonlinear optical properties, and this has led to expedited research in the field of nano‐scale optoelectronic and photonic devices fabrication [3]. Nonlinear optical nanomaterials have the potential to enhance the efficiency and expand the application scope of these techniques to biosensors [4], imaging [5], optical devices [6], communications [7], optical microscopy [8], photocatalysis [9], and optical limiting [10]. This has led to practical efforts to tune the properties of these nanomaterials by size control, morphology engineering, and composition modifications.

Noble metal nanoparticles (NPs), especially silver (Ag) and gold (Au) NPs, are widely studied plasmonic materials because their distinctive properties originate from localized surface plasmon resonance (LSPR). The intense local electromagnetic field generated by LSPR significantly amplifies third‐order nonlinear processes, including reverse saturable absorption (RSA) and nonlinear refraction [11]. The nonlinear optical response of such NPs can be systematically tuned by varying the nanoparticle size [12, 13, 14, 15] and morphology, as well as plasmon coupling with other metal NPs [16, 17]. Z‐scan measurements using nanosecond laser pulses reveal strong reverse saturable absorption and self‐focusing behavior in silver‐doped nanocomposites, with a third‐order susceptibility χ^(3)^ reaching around 10^−13^ esu, accompanied by an increased nonlinear refractive index as Ag loading strengthens local electromagnetic field confinement [18]. LSPR of Ag NPs are highly responsive to the surrounding dielectric environment and interparticle interactions, which makes them attractive candidates for engineering tailored photonic responses. In our previous study, we explored silver‐silver sulfide (Ag‐Ag_2_S)‐decorated multiwalled carbon nanotubes to enhance optical‐limiting performance. The ability of LSPR to improve both linear and nonlinear optical properties has been exploited in surface‐enhanced Raman scattering, photonic waveguides, and ultrafast all‐optical switching [19, 20]. However, many existing materials still struggle to deliver broadband modulation while maintaining stable multiwavelength laser performance, highlighting the need for more advanced and effective material systems to address this issue.

Bimetallic alloy nanomaterials represent a compelling strategy to overcome such limitations with the potential to surpass monometallic counterparts. The incorporation of a second metal can influence, and potentially enhance, the material's optical and magnetic properties. The synergistic interplay in such systems enables the development of multifunctional platforms suitable for diverse photonic applications. Rudenko et al. explored Au@Ag bimetallic core–shell NPs dispersed in glass hosts and found that they exhibit a pronounced surface plasmon resonance absorption band and a strong nonlinear optical response when irradiated with nanosecond laser pulses. This revealed a variety of nonlinear optical mechanisms that depend on composition [19]. Similarly, Yan et al. reported that the nonlinear absorption coefficient of Indium Tin Oxide composite films incorporating Au@Ag bimetallic NPs can be regulated across a wide range, with a transition from saturable to reverse saturable absorption as the bimetallic layer thickness increased [19, 20]. Recently, bimetallic nanowire‐integrated MoS_2_ has demonstrated significant two‐photon absorption (TPA), leading to enhanced optical limiting performance, with an optical limiting threshold that outperforms many previously reported materials [21]. In bimetallic systems, interfacial electronic coupling between constituent metals can significantly modify charge carrier dynamics, facilitating excited‐state absorption (ESA) and enhancing RSA. These systems are expected to exhibit intensity‐dependent nonlinear absorption behavior arising from excited‐state dynamics. Silver‐cobalt (AgCo) nanomaterials combine the strong plasmonic response of Ag with the electronic and magnetic characteristics of Co, offering a promising platform for tuning light–matter interactions. Parang et al. reported the photophysical properties of Ag/Co NPs prepared by chemical reduction, which exhibit a distinct fluorescence emission peak that shifts from ∼485 nm for Ag NPs to ∼538 nm for Ag/Co NPs, with a progressive red shift of the emission with increasing excitation wavelength [22]. This shift in emission indicates modifications in the electronic structure upon cobalt incorporation, which are directly relevant to the excited‐state dynamics that govern the nonlinear optical response of the bimetallic system. In Ag‐Co systems, the partial oxidation of cobalt at the surface introduces defect states and facilitates interfacial charge transfer, both of which play crucial roles in excited‐state absorption and nonlinear optical responses of the system.

Plasmon‐assisted hot‐carrier injection at metal–semiconductor Schottky junctions, such as Ag‐TiO_2_ [11], Ag‐MoS_2_ [23], Ag‐CeO_2_ [24], and Au‐CuO‐type interfaces [25], has been widely exploited to enhance third‐order nonlinear optical (NLO) responses. However, in these systems, the injected carriers are typically accommodated within a conduction‐band continuum or shallow defect/oxygen‐vacancy states in a single‐valence oxide, and the resulting enhancement is governed primarily by plasmonic field amplification and interfacial charge‐separation efficiency. The specific role of an oxide possessing an intrinsic *mixed‐valence* electronic structure, offering discrete sub‐bandgap intermediate levels rather than a structureless continuum, in governing the excited‐state absorption pathway remains largely unexplored. Co_3_O_4_ is distinguished among transition metal oxides by its mixed‐valence spinel structure, in which Co^2+^ and Co^3+^ ions occupy tetrahedral and octahedral sites, respectively, giving rise to d–d and ligand‐to‐metal charge‐transfer (LMCT) transitions at sub‐bandgap energies. Here, Ag‐Co bimetallic nanoparticles incorporating a surface Co_3_O_4_ layer were synthesized, and their third‐order nonlinear optical response was systematically investigated under continuous‐wave and nanosecond excitation.

## 2 Materials and Methods

### 2.1 Chemicals Used

Chemicals used in this study, including silver nitrate, sodium borohydride, cobalt nitrate hexahydrate, and polyvinylpyrrolidone (PVP), were procured from the same suppliers and purity grades as in our previous work [26]. In addition, sodium hydroxide (NaOH) pellets (extrapure, ≥ 98%) were procured from Finar Chemicals Ltd., India. No additional purification was performed on the chemicals, and Milli‐Q water was used to prepare all solutions.

### 2.2 Synthesis of AgCo Bimetallic NPs (ACO BNPs)

ACO BNPs were synthesized via a two‐step chemical reduction method adopted from our previous study [26]. This method was selected to enable sequential nucleation, wherein Co NPs were first formed and stabilized before the controlled reduction and growth of Ag. This sequential approach minimizes the preferential reduction of Ag^+^ that typically occurs during simultaneous co‐reduction because of the large difference in the standard reduction potentials of Ag^+^/Ag and Co^2+^/Co. Furthermore, the alkaline medium promoted the efficient reduction of Co^2+^ by NaBH_4_, thereby facilitating the formation of metallic Co nanoparticles prior to silver deposition.

First, PVP (2.5 mM) was dissolved in deionized water (30 mL) under constant stirring to form a homogeneous polymer solution which served as a stabilizing agent. Several drops of NaOH (2 mM) were added, and the mixture was stirred until fully dissolved, creating an alkaline environment that promotes the effective reduction of Co^2+^ ions and ensures controlled nucleation of nanoparticles. Co(NO_3_)_2_·6H_2_O (1 mM) was dissolved in deionized water (5 mL) and added dropwise to the PVP‐NaOH solution. Simultaneously, NaBH_4_ (5 mL of 10 Mm) solution was introduced dropwise under continuous stirring, serving as a reducing agent. The gradual color transition from colorless to pale gray confirmed the successful formation of cobalt nanoparticles (Co NPs) that were stabilized within the PVP matrix.

In the second step, AgNO_3_ (1 mM) dissolved in deionized water (15 mL) was added dropwise to the as‐prepared Co NPs dispersion under stirring at room temperature, along with NaBH_4_ (2 mM) in deionized water (2 mL) introduced simultaneously to facilitate the controlled reduction of Ag^+^ ions. The solution color progressively transitioned from pale gray to dark brown, confirming the nucleation and growth of Ag species in association with the Co nanoparticles and indicating the successful formation of ACO BNPs.

### 2.3 Z‐Scan and Optical Limiting With CW Laser Excitation

A conventional Z‐scan configuration [27] was employed to investigate the nonlinear optical response and optical limiting characteristics of the nanoparticles using a continuous‐wave diode laser with a wavelength of 638 nm. The laser beam was focused using a 50 mm focal‐length convex lens, and the nanocomposite dispersion placed in a 1 mm cuvette was mounted on a computer‐controlled linear translation stage near the focal region. The Rayleigh range corresponding to the focused beam (∼3.34 mm) exceeded the cuvette thickness, confirming that the measurements satisfied the thin‐sample criterion. The transmitted light intensities at different sample positions were recorded using a Thorlabs S121 C power sensor coupled to a PM5020 power meter. Furthermore, intensity‐dependent transmission studies were conducted to evaluate the optical limiting behavior of the thin films.

### 2.4 Z‐Scan and Optical Limiting With Nanosecond Laser Excitation

Open‐aperture Z‐scan measurements with nanosecond excitation were performed using a Q‐switched Nd: YAG laser producing 5 ns pulses at 532 nm with an incident intensity of $4\times 10^{9}\;{W/\text{cm}}^{2}$. The sample's linear transmittance at this wavelength was adjusted to 56%. The dispersion was contained in a 1 mm cuvette mounted on a computer‐controlled translation stage and translated along the *Z*‐axis through the focal region of a plano‐convex lens with a focal length of 20 cm. The focused beam had a waist of approximately 18 μm, which corresponded to a Rayleigh length of 1.9 mm. The transmitted pulse energy at each Z position was monitored using a pair of pyroelectric detectors in combination with an energy ratio meter (RjP‐735 and RjP‐7620, Laser Probe Inc).

Before Z‐scan measurements on the studied samples, the experimental setup was calibrated using a standard carbon disulfide (CS2) sample. The measured nonlinear optical response of CS_2_ agreed well with reported literature values, thereby validating the alignment, beam focusing, and overall performance of the Z‐scan setup.

### 2.5 Degenerate Four‐Wave Mixing (DFWM) Technique

Four‐wave mixing is a third‐order nonlinear optical process in which four electromagnetic waves interact through a material's χ^(3)^ polarization. When all the participating waves have the same frequency, the process is referred to as degenerate four‐wave mixing (DFWM). Typical DFWM arrangements include phase‐conjugate and BOXCARS geometries. In this study, we adopted a forward‐folded BOXCARS configuration. In this setup, the incident laser beam was divided into three components, which were directed to form the three vertices of a square. The two pump beams, placed on opposite sides, had equal intensities, whereas the third beam, the probe, was adjusted to approximately 20% of the pump intensity. When these three beams overlap spatially and temporally within the sample, a fourth beam (known as the signal beam) is generated at the remaining corner of the square due to nonlinear wave mixing, and this beam is subsequently detected [28].

For the DFWM measurements, plane‐polarized 7 ns laser pulses at 532 nm, produced by the second harmonic of a Q‐switched Nd: YAG laser (Quanta Ray, Spectra Physics), were employed. A polarizer controlled the intensity of the incident beam, and a pyroelectric energy sensor monitored the input pulse energy. The signal beam in the far field was recorded using a calibrated photodiode. All interacting beams had a diameter of approximately 8 mm and were employed without additional focusing. Calibration of the setup was performed using CS_2_, yielding a *χ*_yxyx_/*χ*_xxxx_ ratio of 0.695, which closely matches the reported value of 0.706 [29].

### 2.6 Characterizations

Surface morphology was studied using High‐Resolution Transmission Electron Microscopy coupled with Energy‐Dispersive X‐ray Spectroscopy (EDS) (HRTEM; JEOL Japan; JEM‐2100 Plus). Structural studies were performed using X‐ray diffraction (XRD; Rigaku Ultima IV). Rietveld refinement of the XRD patterns was carried out using the GSAS‐II software package [30]. The optical properties were measured using a SHIMADZU‐1900i UV‐visible spectrophotometer. X‐ray Photoelectron Spectroscopy (XPS) measurements were carried out using a Thermo Scientific K‐Alpha system and the analysis was performed using KherveFitting software [31].

## 3 Results and Discussion

### 3.1 XRD Study

The crystallographic structure and phase composition of the synthesized ACO BNPs were first examined using XRD, and the resulting patterns are presented in Figure 1a. The diffraction profile of the ACO BNPs displayed four well‐resolved reflections at 2*θ* = 38.82°, 45.10°, 65.20°, and 78°, corresponding to the (111), (200), (220), and (311) planes of the face‐centered cubic (fcc) silver lattice, respectively, in agreement with the ICDD reference pattern for Ag. The reference pattern for cobalt (ICDD 00‐015‐0806) is also included in Figure 1a, and no reflections attributable to metallic cobalt, CoO, or Co_3_O_4_ were detected in the experimental pattern of the ACO BNPs. The pure Ag NPs pattern serves as a reference and displays peaks at 37.90°, 44.08°, 64.30°, and 77.30°. A systematic blue shift was observed for all four reflections in the ACO BNPs. According to Bragg's law, a shift toward higher 2θ values corresponds directly to a contraction of the interplanar d‐spacing and, therefore, a reduction in the fcc lattice parameter relative to pure Ag (*a*_0_ = 4.086 Å). Cobalt has a significantly smaller atomic radius (125 pm) than Ag (144 pm), and its incorporation into the Ag lattice is expected to introduce local compressive strain, resulting in this systematic peak displacement [32]. Rietveld refinement of the diffraction patterns was performed using GSAS‐II software (Supporting Information S1: Figure S2 and Table S1) [30], yielding refined lattice parameters of *a* = 4.088 Å for pure Ag NPs and *a* = 4.08712 Å for Ag‐Co BNPs, both crystallizing in the cubic Fm‐3m space group. The difference curves (*I*_exp_–*I*_cal_) indicate good agreement between the experimental and calculated profiles, with weighted profile R‐factors (*R*_wp_) of 6.542% and 4.506% for the Ag NPs and AgCo BNPs, respectively (Supporting Information S1: Table S1). Compared with the pure Ag NPs, the AgCo BNPs exhibit a slight decrease in the lattice parameter (Δa = −0.00088 Å), consistent with the observed shift of the Ag diffraction peaks toward higher diffraction angles. Considering the low Co content (∼0.55 wt%) determined by EDS, this small lattice contraction is more reasonably attributed to interfacial compressive strain associated with surface Co‐containing species rather than extensive substitution of Co into the Ag lattice [33]. This interpretation is further supported by the absence of crystalline Co or Co_3_O_4_ reflections in the XRD pattern and the presence of Co^2+^/Co^3+^ species identified by XPS. The complete absence of cobalt or cobalt oxide reflections in Figure 1a is consistent with the presence of cobalt species either in an amorphous state or as nanocrystalline domains below the XRD coherence length (typically > 3–5 nm for Scherrer‐detectable phases) [34]. Co at the nanoparticle surface is present in an oxidized form. An amorphous or sub‐nanometer Co_3_O_4_ surface oxide layer of this type is well documented to be undetectable by XRD while remaining clearly resolved by surface‐sensitive spectroscopic methods [35]. The (111) peak FWHM of 0.995° reflects contributions from both crystallite size broadening and lattice strain, consistent with the nanoscale dimensions confirmed by TEM analysis. The systematic shift of Ag diffraction peaks toward higher 2θ values indicates lattice contraction, suggesting incorporation of smaller Co atoms into the Ag lattice. Although distinct Co phases were not detected by XRD, this behavior is consistent with the formation of an Ag‐rich alloy.

**FIGURE 1:**
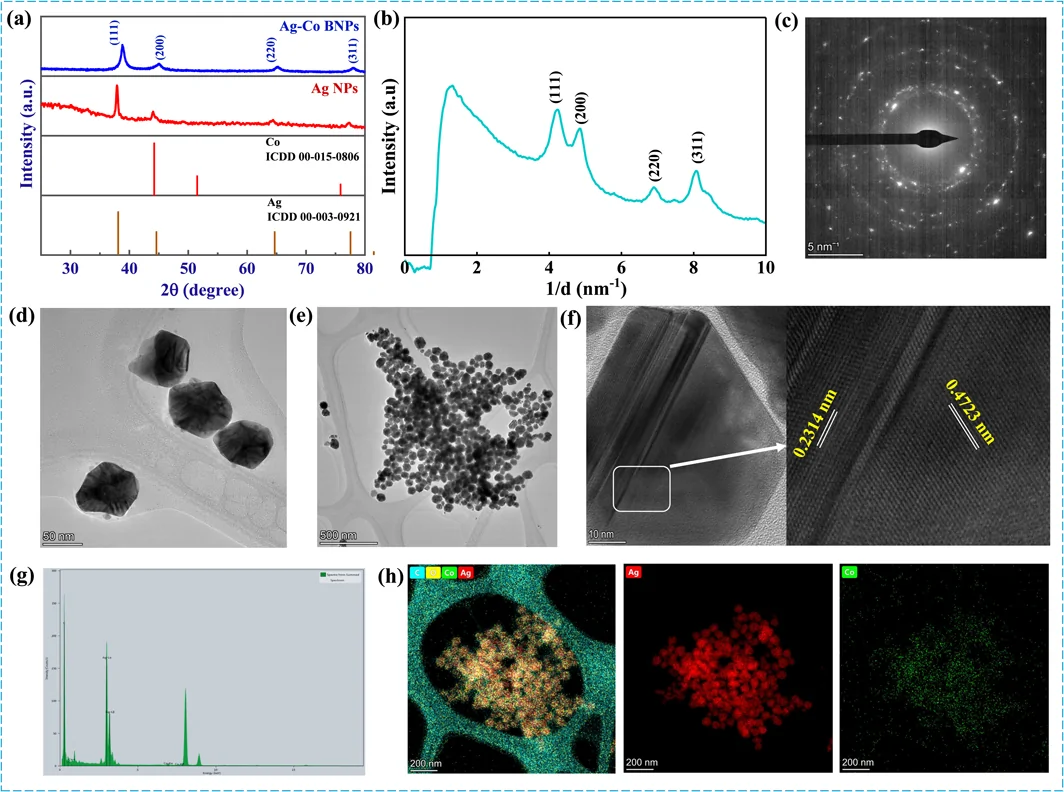
(a) XRD patterns; (b, c) radial intensity profile and corresponding SAED pattern; (d, e) TEM images; (f) HRTEM image showing lattice fringes; (g) EDS spectrum; and (h) elemental mapping confirming the uniform distribution of Ag and Co in ACO BNPs.

### 3.2 Morphological Analysis

The morphology and size characteristics of the ACO BNPs were evaluated by TEM, and the images at two magnifications are shown in Figure 1d,e. The NPs appeared as large, dark, electron‐dense aggregates with a broadly quasi‐spherical shape. Individual NPs within the agglomerated clusters were resolved as quasi‐spherical NPs with generally smooth outer boundaries and moderate size variation. Quantitative analysis of the particle diameters from Figure 1e yielded a mean size of 48.12 nm with a standard deviation of 34.59 nm, suggesting polydispersion. The particle size distribution of the NPs is provided in Supporting Information S1: Figure S1.

This polydispersity has direct and quantifiable consequences for the optical response of an ensemble. Due to the strong size dependence of LSPR in Ag nanoparticles, variations in particle size led to a red shift and broadening of the plasmon band, giving rise to an inhomogeneously broadened absorption profile [36]. Although this reduces the peak extinction at any single wavelength relative to a monodisperse system, it simultaneously extends the spectral range over which the material interacts with light, which may be advantageous for broadband nonlinear optical limiting applications.

Plasmonic coupling between proximate nanoparticles within the agglomerates generates strongly enhanced localized electromagnetic fields in the nanogaps between particles (electromagnetic hotspots) through near‐field hybridization of individual LSPR modes, producing field enhancement factors that can exceed 10^4^ in sub‐nanometer gaps [37, 38, 39]. Since third‐order nonlinear optical processes scale with the square of the local field intensity, even a modest population of hotspot‐bearing agglomerates can disproportionately amplify the nonlinear response of the ensemble [40], consistent with recent experimental observations of proximity‐dependent nonlinear absorption enhancement in coupled plasmonic nanoparticle assemblies [41].

High‐resolution TEM imaging was performed to directly resolve the atomic‐scale crystal structure of individual BNPs, and the resulting HRTEM image with a magnified inset showing the resolved lattice fringes is presented in Figure 1f. Two distinct fringe spacings were resolved and measured directly from the image in Figure 1f: a dominant spacing of 0.231 nm and a secondary spacing of 0.4723 nm. The fringe spacing of 0.2314 nm agrees with the (111) interplanar spacing of fcc Ag (d_111_ = 0.2359 nm). This assignment confirms the presence of well‐ordered crystalline silver in the particle interior, consistent with the XRD and SAED results shown in Figure 1a–c.

The secondary fringe spacing of 0.4723 nm, observed in localized regions proximal to the particle surface (Figure 1f), does not correspond to any principal reflection of metallic Ag, metallic Co, or CoO. This value closely matches the (111) interplanar spacing of cubic spinel Co_3_O_4_ (d_111_ = 0.4666 nm) [42]. The spatial localization of this fringe spacing to the surface‐proximal regions of the nanoparticle, rather than the crystalline interior, is physically consistent with a thin surface oxide layer formed by the preferential oxidation of surface cobalt atoms.

The elemental composition and spatial distribution of the constituent elements in the ACO BNPs were assessed using energy‐dispersive X‐ray spectroscopy, with the EDS spectrum shown in Figure 1g and the corresponding elemental maps presented in Figure 1h. The EDS spectrum shows peaks attributable to Ag, Co, O, and C, with the dominant Ag signals clearly visible and the Co signal at low intensity. Semi‐quantitative analysis of the measured region yielded approximately 58.1 wt. %, 0.55 wt. %, 1.25 wt. %, and 40.1 wt. % for Ag, Co, O, and C, respectively. The carbon signal is attributed to the PVP capping agent used during synthesis and the carbon support film of the TEM grid and is not intrinsic to the nanoparticle composition.

Elemental mapping in Figure 1h provides spatial resolution of these compositional findings. Ag was distributed broadly and intensely throughout the particle volume, consistent with its role as the dominant crystalline phase. In contrast, cobalt appears at a significantly lower signal intensity and is distributed diffusely across the particle without being resolved as a discrete geometrically defined shell.

The cobalt content of ∼0.55 wt% relative to silver is low; however, at such dilute concentrations, cobalt atoms can be incorporated into the Ag lattice to form an Ag‐rich alloy core. The absence of distinct cobalt or cobalt oxide phases in the XRD patterns, together with the observed lattice contraction, supports this alloying behavior. Simultaneously, thermodynamic factors favor the surface segregation of cobalt because the lower surface energy of Ag relative to Co drives the preferential migration of cobalt toward the nanoparticle surface. This leads to the formation of a cobalt‐enriched surface region, which is further stabilized through oxidation to Co_3_O_4_, as confirmed by XPS. Such coexistence of an alloyed core with a surface‐segregated oxide layer is commonly observed in bimetallic nanoparticles with large surface energy differences [33].

### 3.3 XPS Analysis

X‐ray photoelectron spectroscopy was performed to establish the oxidation state and chemical environment of each constituent element at the nanoparticle surface (Supporting Information S1: Figure S2), exploiting its surface sensitivity (sampling depth ∼5–10 nm) to probe the surface‐proximal region, which is inaccessible to bulk techniques such as XRD.

The Ag 3d spectrum exhibits two well‐resolved peaks at 368.19 eV (3d_5/2_) and 374.10 eV (3d_3/2_), with a spin–orbit splitting of 5.91 eV. The 3d_5/2_ binding energy of 368.19 eV falls within the established range for metallic silver (Ag^0^: 367.9–368.4 eV) as tabulated in the XPS database, confirming that silver retains its fully metallic character throughout the nanoparticle and is not subject to surface oxidation under the employed synthesis conditions. This result is thermodynamically consistent with the high standard reduction potential of the Ag^+^/Ag^0^ couple (+0.80 V vs. SHE) and the well‐established resistance of metallic silver to oxidation under ambient conditions. The spin–orbit splitting value of 5.91 eV is close to the reference value for metallic Ag (6.00 eV).

The Co 2p spectrum presented a more complex multiplet structure, indicative of mixed cobalt oxidation states at the nanoparticle surface. Peaks were observed at 780.61 and 782.27 eV in the Co 2p_3/2_ region, accompanied by satellite features at 785.91 eV and Co 2p_1/2_ components at 795.75 and 797.49 eV. These spectral features are interpreted considering the Co 2p fitting parameters compiled by Biesinger et al. [35], which establishes that Co_3_O_4_ produces Co 2p_3/2_ components spanning approximately 780.2–783.1 eV, with a satellite feature at ∼787 eV. These values correspond closely to those measured in this study. Co 2p_3/2_ peaks at 780.61 and 782.27 eV correspond to the Co^3+^ and Co^2+^ species, respectively, consistent with the mixed‐valence spinel Co_3_O_4_ phase. The pronounced satellite peak at ∼785.91 eV further confirmed the presence of Co^2+^, a characteristic feature of Co_3_O_4_. The Co 2p_1/2_ peaks at 795.75 and 797.49 eV correspond to the expected spin‐orbit splitting of cobalt oxides. Importantly, the absence of any feature near ∼778 eV confirms that metallic Co is not present, indicating that cobalt predominantly exists in an oxidized state.

The O 1s spectrum shows two components at 531.16 and 532.62 eV, which are attributed to lattice oxygen (M–O) and surface hydroxyl/adsorbed oxygen species, respectively. The absence of a lower‐binding‐energy component (< 530 eV) indicates that the oxide is primarily surface‐confined and lacks bulk crystallinity, consistent with a thin Co_3_O_4_ layer.

### 3.4 Linear Optical Properties

The UV–Vis absorption spectrum of the Ag NPs displays a well‐defined localized LSPR peak at 406 nm, consistent with the coherent oscillation of conduction‐band electrons in quasi‐spherical Ag NPs and in agreement with the values reported for PVP‐stabilized Ag NPs of comparable dimensions [36, 43]. As shown in Figure 2a, upon cobalt incorporation, the LSPR band red‐shifted substantially to ∼510 nm with concurrent broadening and attenuation of the peak intensity. This spectral evolution reflects the superposition of several interdependent effects. The presence of Co and the formation of AgCo heterostructures modified the electronic structure and effective dielectric response of Ag. The partially filled Co 3d states introduce additional interband electronic transitions, enhance electron‐phonon scattering, and alter the effective dielectric function and conduction electron response of the Ag NPs. These collectively lead to the damping and red shift of Ag LSPR [44, 45].

**FIGURE 2:**
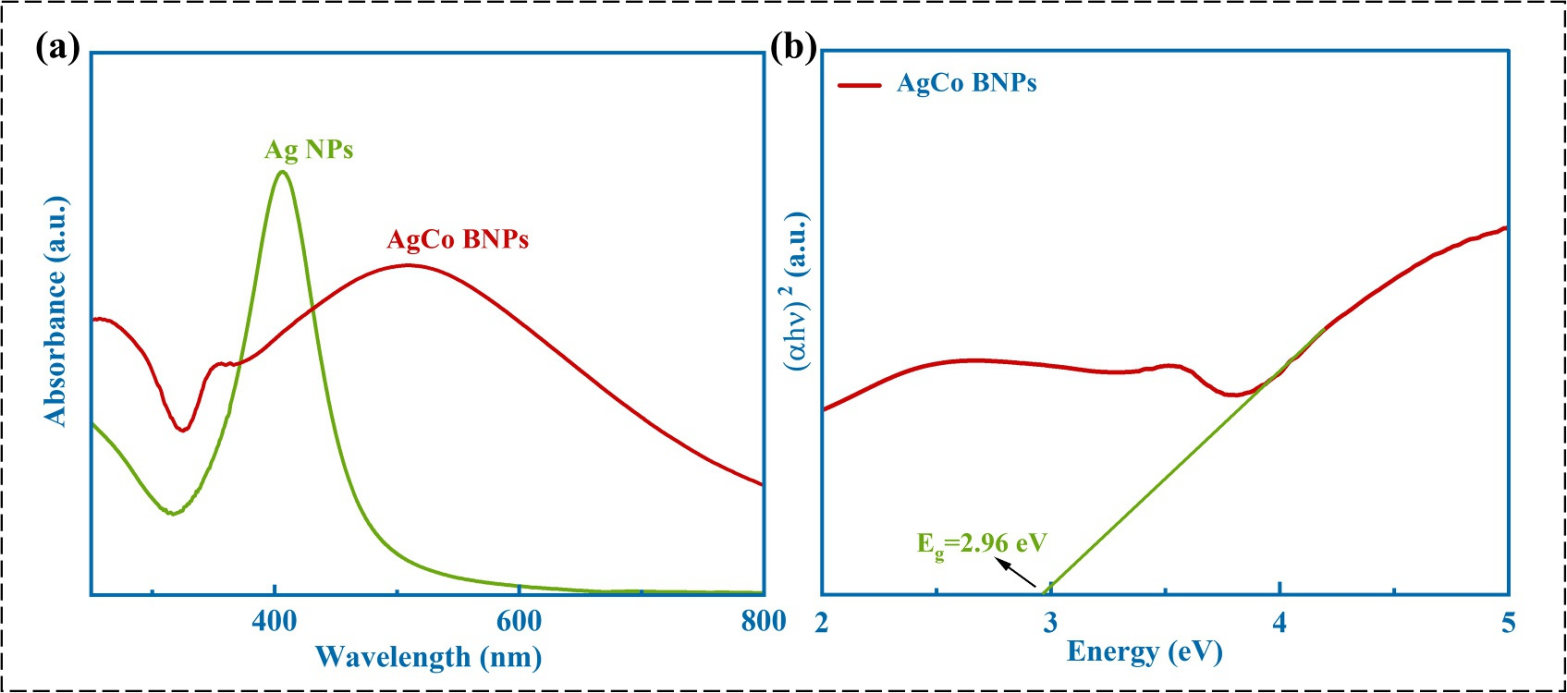
(a) UV‐Visible absorption spectra of Ag NPs and AgCo BNPs. (b) Tauc plot ${\left(\alpha h\nu \right)}^{2}$ versus photon energy.

This behavior has been previously reported for such systems and is consistent with the red shift observed in our system from 406 to ∼510 nm [46, 47]. Furthermore, interfacial charge redistribution at the Ag‐Co_3_O_4_ interface modifies the electronic structure of Ag and facilitates hot‐carrier transfer across the metal‐semiconductor junction. A similar interfacial charge transfer has been demonstrated for Ag‐Co_3_O_4_ heterostructures through first‐principles calculations [48].

The polydisperse size distribution (Supporting Information S1: Figure S1) and interparticle agglomeration observed in TEM further contribute to inhomogeneous broadening of the LSPR band and promote near‐field plasmonic coupling between adjacent NPs [49]. In addition, the broad visible absorption extending beyond 600 nm further suggests a contribution from Co_3_O_4_ ligand‐to‐metal charge transfer (LMCT) transitions, which are known to produce absorption bands in the 400–550 nm region [50]. These structural and electronic features collectively shape the linear optical response and provide the basis for the enhanced nonlinear optical behavior of ACO BNPs.

In addition to modifying the linear optical spectrum, these electronic interactions are directly relevant to the nonlinear optical response. The Ag LSPR generates intense localized electromagnetic fields that substantially increase the excitation probability of the adjacent Co_3_O_4_ surface states [40]. In addition, theoretical studies have shown that maintaining a low cobalt concentration preserves the strong Ag plasmonic field while avoiding excessive plasmon damping, thereby maximizing the local electromagnetic field at the cobalt‐containing regions [51]. This is consistent with the low Co content (∼0.55 wt%) in ACO BNPs, which preserves the strong Ag LSPR while providing sufficient Co^2+^/Co^3+^ intermediate states for charge transfer. Consequently, plasmon‐generated hot carriers are efficiently injected into these intermediate states, increasing the excited‐state population and extending carrier lifetimes [52]. Because these excited states possess a larger absorption cross‐section than the ground state, they promote ESA, resulting in RSA [7].

Although cobalt possesses intrinsic ferromagnetic characteristics arising from its partially filled 3d orbitals, its direct contribution to the observed nonlinear optical enhancement is considered secondary. Instead, the enhanced third‐order nonlinear optical response is primarily governed by the electronic effects of Co, including the modification of the Ag dielectric response, interfacial charge transfer across the Ag‐Co_3_O_4_ heterojunction, and the formation of Co^2+^/Co^3+^ intermediate states that facilitate excited‐state absorption.

The optical bandgap of the ACO BNPs was determined from a Tauc plot constructed under the direct‐transition assumption, yielding *E*_*g*_ = 2.96 eV. The corresponding Tauc plot is shown in Figure 2b. This value significantly exceeds the fundamental optical gap of bulk or crystalline Co_3_O_4_, which is reported to range from 1.5 to 2.4 eV depending on synthesis conditions and nanostructure dimensions [42, 53]. The elevated gap in the present system is attributed to the sub‐nanometer, amorphous character of the Co_3_O_4_ surface layer, where quantum confinement effects raise the effective transition energy above bulk values [42]. Additionally, band bending at the Ag‐Co_3_O_4_ Schottky junction and disorder‐induced defect states at the metal‐oxide interface [54] collectively shift the apparent absorption onset to higher energies. Critically, both excitation wavelengths employed in the NLO measurements (532 nm, 2.33 eV; 638 nm, 1.94 eV) lie below this optical gap, indicating that plasmonic and sub‐gap transitions, rather than band‐to‐band absorption, are responsible for populating the intermediate excited states.

### 3.5 Third‐Order Nonlinear Optical (TNLO) Studies

#### 3.5.1 TNLO With Continuous Wave (CW) Laser Excitation

The open‐aperture Z‐scan technique was used to determine the effective nonlinear absorption coefficient (β_eff_) for each sample. Figure 3a presents the open‐aperture Z‐scan results for ACO at input intensities of $4.7\times 10^{3}$, $5.6\times 10^{3}$, and $6.5\times 10^{3}\;{W/\text{cm}}^{2}$. The characteristic dip observed at the focal position (*Z* = 0) reflects the material's intensity‐dependent nonlinear absorption, which becomes more pronounced at higher excitation levels. The transmittance curves remained symmetric about the focal point, consistent with pure nonlinear absorption behavior. The normalized open‐aperture transmittance can be described using the relation given in [1] as follows:

(1) $$T\left(z\right)=\sum_{m=0}^{\infty }\frac{{\left(-q_{0}\right)}^{m}}{{\left(m+1\right)}^{\frac{3}{2}}}$$

where $q_{0}=\frac{\beta_{\text{eff}}I_{0}\left(1-\exp^{-\alpha L}\right)}{(1+\frac{Z^{2}}{Z_{0}^{2})})\alpha }$. Here, α denotes the linear absorption coefficient, L represents the sample thickness, and $Z_{0}$ is the Rayleigh length. $\beta_{\text{eff}}$ for ACO was extracted by fitting the experimental open‐aperture Z‐scan data to Equation (1). The resulting $\beta_{\text{eff}}$ values at various input intensities are summarized in Table 1. A comparison with previously reported nonlinear‐absorbing materials is provided in Table 2, demonstrating that the synthesized nanoparticles exhibit enhanced nonlinear optical performance relative to many existing systems.

**FIGURE 3:**
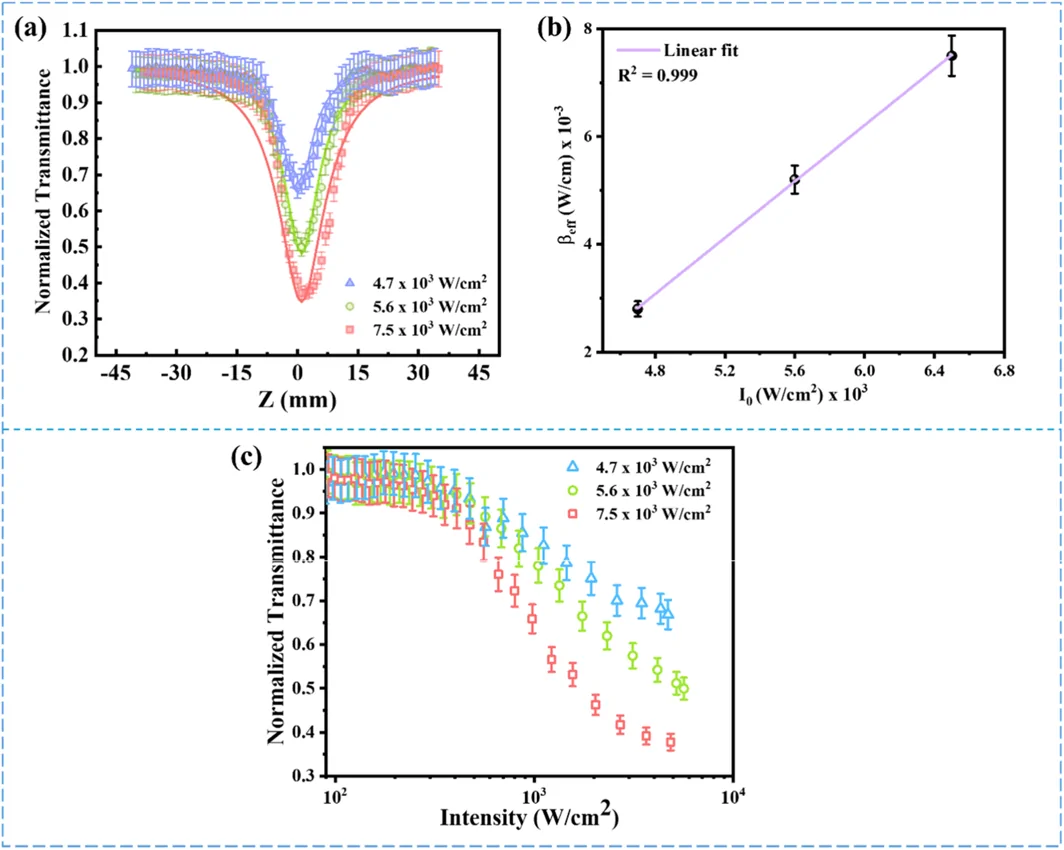
(a) Open aperture Z‐scan curves of ACO at different on‐axis intensities (The solid lines depict the theoretical fit). (b) Variation of β_eff_ with input intensity (I_0_). (c) Normalized optical transmittance as a function of input intensity of ACO.

**TABLE 1:** Third‐order nonlinear optical coefficients of ACO.

Intensity (W/cm^2^) $\times \;10^{3}$	β_eff_ (cm/W) $\times \;10^{-3}$	*I*_on_ (W/cm^2^)
4.7	2.8	570
5.6	5.2	477
6.5	7.5	355

**TABLE 2:** A comparison of various materials employed and their individual NLO results.

Sl. No.	Samples	β_eff_ (cm/W)	Reference
1.	Au@Ag bimetallic NPs	1–5 × 10^−4^	[13]
2.	Gold nanoparticle colloids capped with gum Arabic	4.46–13.3 × 10^−4^	[55]
3.	Ni/Cu/Ag trimetallic NPs	4.12 × 10^−5^	[56]
4.	VSe_2_‐CoSe_2_ NPs	1.25 × 10^−4^	[57]
5.	ACO BNPs	4.4–10.5 × 10^−3^	Present work

Nonlinear absorption (NLA) in a material can arise from several mechanisms, such as RSA, TPA, and ESA, depending on factors such as linear absorption, excitation wavelength, and incident beam intensity. The characteristic dip observed in the open‐aperture Z‐scan confirms the presence of RSA in nanoparticles. Figure 3b presents the variation of $\beta_{\text{eff}}$ with input intensity. If the NLA originated purely from TPA $\beta_{\text{eff}}$ would remain independent of the excitation intensity. In contrast, a clear linear increase in $\beta_{\text{eff}}$ with intensity is observed, indicating that the process cannot be explained by TPA alone. Instead, this behavior is consistent with a sequential TPA mechanism accompanied by excited‐state absorption, leading to an ESA‐assisted RSA response [13, 28].

At elevated pump intensities, the sample exhibited a marked reduction in transmission, confirming the presence of NLA, which is a key requirement for efficient optical limiting applications. An ideal optical power limiter (OPL) maintains linear transmission at low incident powers but becomes increasingly opaque as the input intensity rises [7, 29], thereby safeguarding sensitive optical components and the human eye from high‐power laser exposure. The OPL characteristics extracted from the open‐aperture Z‐scan data are shown in Figure 3c. Although the transmission initially follows Beer's law, noticeable deviations appear once the input intensity exceeds a threshold. The normalized transmittance of ACO BNPs as a function of input intensity was analyzed to assess its optical limiting performance and to determine the onset intensity $I_{\text{on}}$ (the point where limiting begins). A lower $I_{\text{on}}$ signifies a more effective optical limiter [13, 58]. The OPL behavior of ACO BNPs is presented in Figure 3, and the corresponding $I_{\text{on}}$ value is listed in Table 1.

#### 3.5.2 TNLO With Nanosecond Pulsed Laser Excitation

The open‐aperture (OA) Z‐scan curves for the samples are shown in Figure 4a. The plots display symmetric intensity‐dependent transmission, with a pronounced dip at the focal position (*Z* = 0). As the incident laser intensity increased, the transmittance decreased, confirming the presence of strong RSA in the materials. The experimental results were fitted numerically using the TPA model described in the corresponding Equation (2).

(2) $$T=\left({\left(1-R\right)}^{2}\exp\left(-\alpha L\right)/\sqrt{\pi }q_{0}\right)\int_{-\infty }^{+\infty }\ln\left[\sqrt{1+q_{0}\;\exp\left(-t^{2}\right)}\right]dt.$$

In this context, *T*, *L*, and *R* denote the sample transmission, sample thickness, and surface reflectivity, respectively. Parameter α corresponds to the linear absorption coefficient. The on‐axis peak intensity is represented by $I_{0}$, and the parameter $q_{0}$ is obtained from the expression $\beta_{\text{eff}}\left(1-R\right)I_{0}L$, where $\beta_{\text{eff}}$ is the effective TPA coefficient. The effective path length $L_{\text{eff}}$ is calculated using L=[1−exp(−αL)]/α. Based on this analysis, the two‐photon absorption coefficient of the NPs was determined to be 0.054 m/GW. Table 3 compares the present system with previously reported materials under similar excitation conditions. Although the *β* value is comparable to that of several reported systems, the ACO BNPs exhibited a balanced and enhanced nonlinear optical performance when considering both the magnitude of nonlinear absorption and optical limiting behavior. In particular, the relatively low optical limiting threshold, along with the experimentally validated excited‐state absorption mechanism and plasmon‐assisted enhancement, highlights the advantages of the present system over conventional single‐component materials.

**FIGURE 4:**
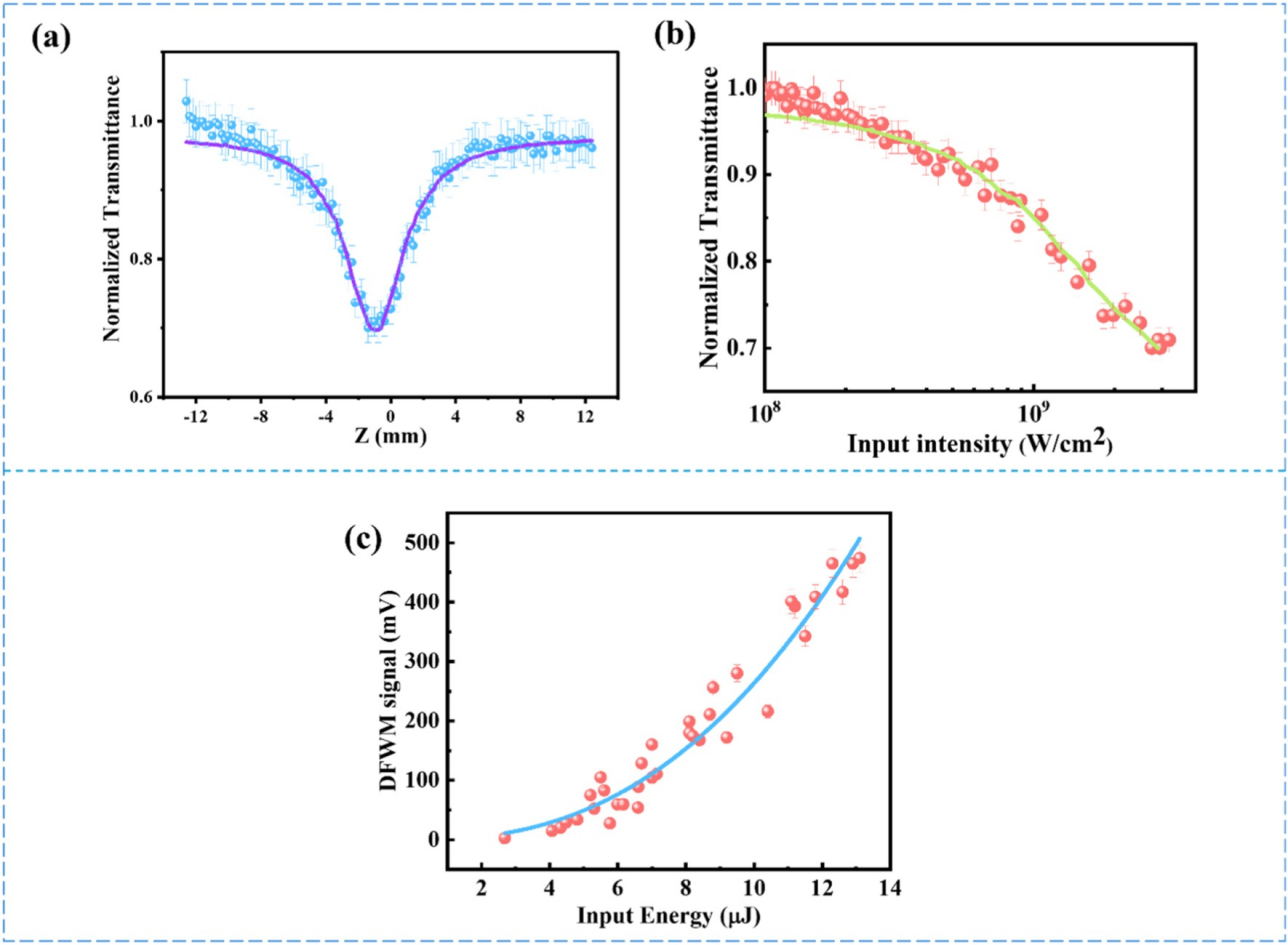
(a) Open‐aperture Z‐scan plots of ACO BNPs (solid lines are theoretically fitted to the experimental data). (b) Nonlinear optical transmission as a function of input intensity (Optical Limiting) in ACO BNPs. (c) DFWM signal of ACO BNPs (squares are data points, whereas the solid curve is a cubic fit to the data).

**TABLE 3:** Comparison of the nonlinear absorption coefficient (β) of ACO BNPs with previously reported materials under 532 nm excitation.

Sl. No.	Samples	β (m/GW)	Reference
1.	ZnO/MWCNTs	0.0064	[59]
2.	Non‐covalently functionalized rGO	0.28	[60]
3.	ZnTNP‐PAES	0.05282	[61]
4.	MWNTs‐OH	0.105	[62]
5.	ACO BNPs	0.054 (5.4 × 10^−9^ cm/W)	Present work

The optical limiting (OL) behavior of the samples was extracted from the open‐aperture Z‐scan data and represented as normalized transmittance (NT) versus incident laser intensity. Optical limiting is a key feature of nonlinear optical materials because it attenuates high‐intensity laser radiation and helps safeguard sensitive optical devices and human vision [7, 57]. The OL responses of the samples are shown in Figure 4b. A decrease in the normalized transmittance from 100% to 69% was observed as the input intensity increased, confirming the strong limiting action. The onset of optical limiting for the sample occurred at $2\times 10^{8}\;{W/\text{cm}}^{2}$. This reduction in transmission arises from pronounced nonlinear optical interactions within the material. These results highlight the suitability of the samples for OL applications, including eye and sensor protection applications. The error bars shown in the Z‐scan plots represent the experimental uncertainty associated with laser pulse energy fluctuations, detector noise, and the data acquisition system. The uncertainty in the extracted nonlinear absorption coefficient was determined from the reproducibility of repeated measurements and was found to be approximately 3%–5%.

Due to differences in excitation conditions and nonlinear processes, the nonlinear absorption coefficients obtained with CW and nanosecond laser stimulation cannot be directly compared. Thermal effects from continuous energy input, such as thermal lensing, heat‐induced changes in refractive index, and thermally assisted excited‐state absorption, are included in the reported $\beta_{\text{eff}}$, an effective nonlinear absorption coefficient with CW excitation. Consequently, $\beta_{\text{eff}}$ represents not only the material's intrinsic nonlinear absorption but also its thermal and electronic nonlinearities. On the other hand, because the pulse duration is much shorter than the characteristic heat diffusion time, nanosecond‐pulsed excitation is dominated by fast electronic nonlinearities, such as ESA and RSA, with minimal thermal contribution. So, the nonlinear absorption coefficient *β* measured by the nanosecond open‐aperture Z‐scan method primarily reflects the material's electronic nonlinear response. This difference between CW and pulsed measurements is well known in Z‐scan theory and nonlinear optics research. Therefore, the differences observed between $\beta_{\text{eff}}$ (CW) and *β* (nanosecond) are due to differences in excitation conditions and absorption mechanisms, rather than any inconsistency in the experimental measurements [27, 63].

#### 3.5.3 DFWM Studies

The third‐order nonlinear optical susceptibility of the sample was evaluated using the DFWM technique. Figure 4c shows the variation in the DFWM signal with the input intensity. As expected for a third‐order process, the measured signal scales with the cube of the incident intensity, which is consistent with the relationship described by the following equation:

$$I\left(\omega \right)\;\propto \;{\left(\frac{\omega }{2\epsilon_{0}cn^{2}}\right)}^{2}{\left|\chi^{3}\right|}^{2}l^{2}I_{0}^{3}\left(\omega \right),$$

where I(ω) is the intensity of the DFWM signal, $I_{0}\left(\omega \right)$ intensity of the pump beam, *l* is the sample length, and *n* is the linear refractive index of the sample. The $\chi^{3}$ some of the samples have been determined from the following equation

$${\left|\chi^{\left(3\right)}\right|}_{\text{sample}}={\left|\chi^{\left(3\right)}\right|}_{\text{ref}}{\left(\frac{I_{\text{sample}}}{I_{ref}}\right)}^{\frac{1}{2}}{\left(\frac{n_{\text{sample}}}{n_{\text{ref}}}\right)}^{2}\left(\frac{l_{\text{ref}}}{l_{\text{sample}}}\right)\left(\frac{\alpha_{0}l}{e^{\frac{-\alpha_{0}l}{2}}\left(1-e^{-\alpha_{0}l}\right)}\right).$$

In the above equation, ‘ref’ refers to the standard reference carbon disulfide (CS_2_) under identical conditions. The ${\left|\chi^{\left(3\right)}\right|}_{\text{ref}}$ value of CS_2_ is taken to be 4.5 × 10^−13^ esu. *I*_sample_ and *I*_ref_ are DFWM signal intensities, $n_{\text{sample}}$ and $n_{\text{ref}}$ are their linear refractive index, $l_{\text{ref}}$ and $l_{\text{sample}}$ is the interaction length in the medium, and *α*_*0*_ is the linear absorption coefficient at 532 nm. The third‐order optical susceptibility (χ^(3)^) and figure of merit of the sample were estimated to be 4.12 × 10^−11^ esu and 7.21 × 10^−14^ esu m.

### 3.6 Mechanism of Nonlinear Optical Response

Figure 5 illustrates the proposed plasmon‐assisted ESA mechanism for ACO BNPs. The nonlinear optical response of the Ag‐Co system is governed by a sequential excitation process involving plasmon‐induced charge transfer and excited state absorption. Upon laser irradiation at 532 or 638 nm, the Ag component undergoes LSPR, generating energetic electrons and holes. These hot carriers are partially transferred across the Ag‐Co_3_O_4_ interface into Co^2+^/Co^3+^‐related d‐states and ligand‐to‐metal charge transfer (LMCT) levels associated with the surface oxide layer [64, 65]. This process leads to the population of relatively long‐lived intermediate states.

**FIGURE 5:**
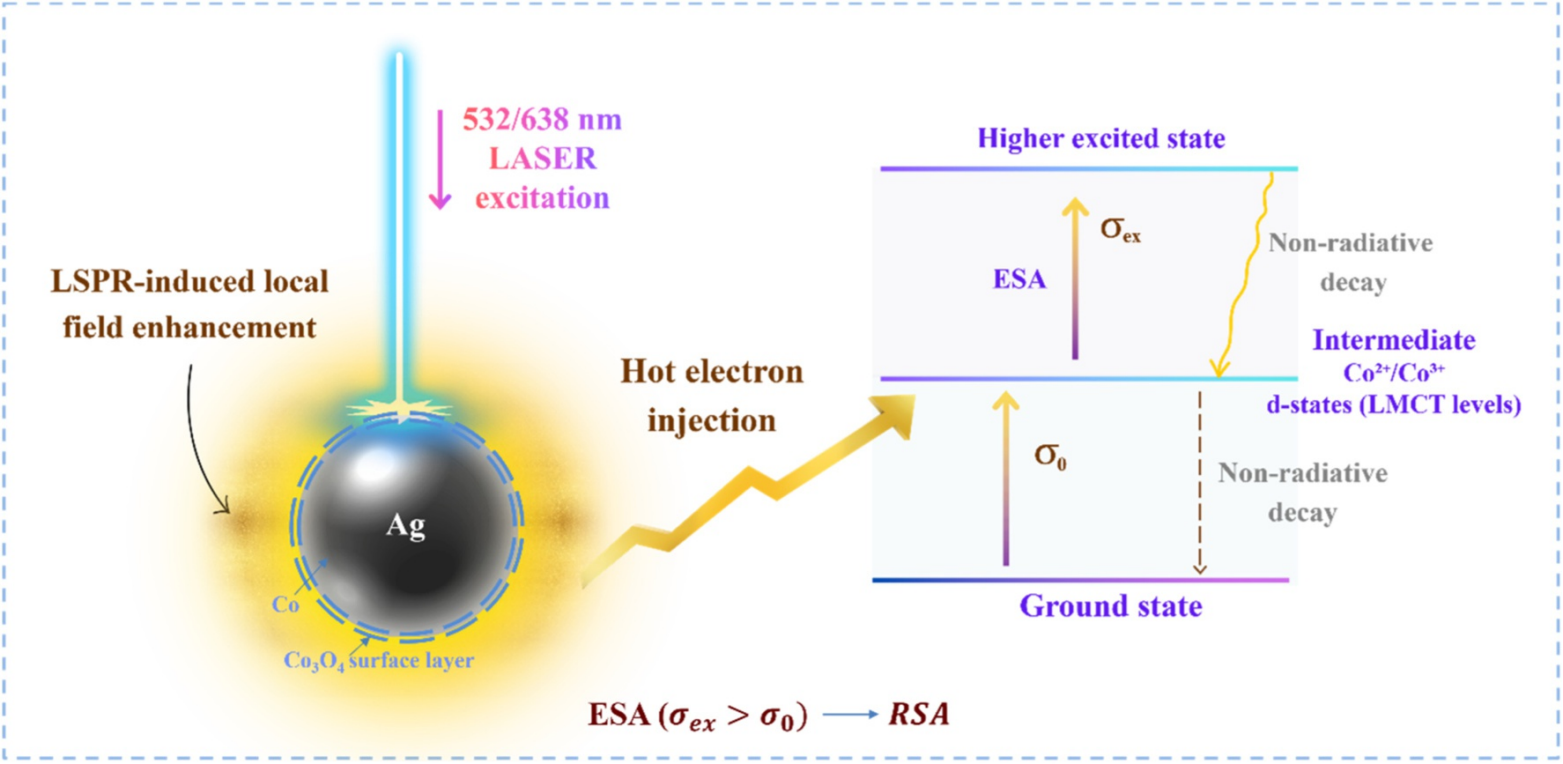
Schematic illustration of plasmon‐assisted ESA mechanism in ACO BNPs.

Simultaneously, Co_3_O_4_ contributes through sub‐bandgap optical transitions, enabling direct excitation into these intermediate levels, as the excitation photon energies are below the optical band gap (∼2.96 eV). The presence of mixed‐valence Co^2+^/Co^3+^ states and interfacial defect levels further increases the density of the accessible intermediate states.

Once populated, these excited states exhibit a higher absorption cross‐section (σ_ex_ > σ_*o*_), satisfying the fundamental condition for RSA. Subsequent photon absorption from these states promotes electrons to higher energy levels, resulting in an ESA‐dominated nonlinear absorption process. The experimentally observed increase in $\beta_{\text{eff}}$ with increasing input intensity confirms this mechanism, distinguishing it from instantaneous two‐photon absorption (TPA), which exhibits an intensity‐independent response [66].

In addition to electronic transitions, the plasmonic Ag core enhances the nonlinear optical response through local electromagnetic field amplification, thereby increasing the effective excitation probability of the Co_3_O_4_ surface states. Furthermore, interparticle interactions and agglomeration can generate localized electromagnetic hotspots, leading to additional field enhancement and strengthening the overall nonlinear absorption [40, 67, 68].

Overall, the nonlinear optical behavior arises from (i) plasmon‐induced local field enhancement, (ii) interfacial charge transfer, and (iii) ESA mediated by the Co_3_O_4_ surface and defect states, resulting in strong RSA and efficient optical limiting.

To position this mechanism relative to other plasmonic metal‐oxide platforms, Table 4, compares representative Ag/Au–oxide Schottky systems reported in the literature with the present Ag‐Co_3_O_4_ system in terms of the junction type, nature of the intermediate electronic states involved, and representative nonlinear optical performance metrics. In the Ag‐TiO_2_, Ag‐MoS_2_, and Ag‐CeO_2_ systems, the injected hot carriers are accommodated within a conduction‐band continuum or shallow defect/oxygen‐vacancy states of a single‐valence oxide, whereas CuO‐based systems lack an analogous multi‐valence electronic structure altogether. The Ag‐ Co_3_O_4_ interface provides discrete sub‐bandgap Co^2+^/Co^3+^ d–d and LMCT intermediate states. We propose that this distinction underlies the sequential ESA process identified above, consistent with the intensity‐dependent increase in $\beta_{\text{eff}}$ (Figure 3)), a signature not explicitly demonstrated in most comparator systems. It should be noted that the nonlinear optical parameters compiled in Table 4 were obtained under different excitation wavelengths, pulse durations, and measurement techniques (single‐beam Z‐scan vs. DFWM); they are therefore presented for qualitative positioning of the present system within the literature rather than as a direct quantitative benchmark.

**TABLE 4:** Comparison of representative plasmonic metal‐oxide heterostructures for third‐order nonlinear optical applications.

System	Junction/Mechanism	Intermediate states	Representative NLO performance	Excitation conditions	Reference
Ag‐TiO_2_ (sputtered film)	Schottky junction; plasmon‐induced hot‐electron injection into TiO_2_	Conduction band (wide‐bandgap semiconductor)	β enhanced 3.4× compared with pristine TiO_2_	fs, 515 nm	[11]
Ag‐MoS_2_	Schottky junction; interfacial charge separation	Conduction band and defect states	*β* = 3.5 × 10^−4^ cm W^−1^	CW, 532 nm	[23]
Ag‐CeO_2_	Schottky junction; plasmon‐assisted hot‐electron injection	Oxygen‐vacancy defect states	Χ^(3)^ = 78.6 × 10^−6^ esu	Pulsed, 1064 nm	[24]
CuO‐based composites *(representative of Au–CuO systems)*	Charge transfer involving single‐valence CuO	Cu^2+^‐related states	*β* ≈ 10^−7^ cm W^−1^; χ^(3)^ ≈ 10^−6^ esu	Q‐switched, 532 nm	[25]
ACO BNPs (present work)	Schottky junction with plasmon‐assisted hot‐electron injection into mixed‐valence Co_3_O_4_	Discrete Co^2+^/Co^3+^ d–d and LMCT intermediate states	$\beta_{\text{eff}}$ = 4.4–10.5 × 10^−3^ cm W^−1^ (CW, 638 nm); χ^(3)^ = 4.12 × 10^−11^ esu (DFWM, 532 nm)	CW (638 nm) and ns (532 nm)	Present work

The pronounced reverse saturable absorption and the low optical limiting threshold highlight the suitability of ACO BNPs for optical limiting applications. The combination of broadband plasmonic absorption and defect‐mediated subgap transitions enables wavelength‐flexible operation. Moreover, the coexistence of plasmonic and interfacial electronic effects provides a platform for tunable nonlinear responses, making these systems promising candidates for photonic switching, laser modulation, and advanced nonlinear optical device applications.

Beyond the present findings, it is worth considering how morphological engineering can further enhance the nonlinear optical response of Ag‐Co nanostructures.

The geometry of metallic nanostructures plays a significant role in modulating these mechanisms. As reported by Torres‐Torres and García‐Beltrán [69], anisotropic morphologies such as nanorods, nanotriangles, and core–shell architectures support additional plasmonic modes and stronger local electromagnetic field enhancement compared with quasi‐spherical NPs, thereby offering greater flexibility for tailoring third‐order nonlinear optical responses. Similarly, cooperative near‐field interactions in nanostructured assemblies can further amplify nonlinear optical effects. These findings suggest that controlled morphological engineering of Ag‐Co nanostructures, through the development of anisotropic or core–shell architectures, represents a promising strategy to further enhance their nonlinear optical performance and extend their applicability in broadband optical limiting and photonic protection devices.

The increased nonlinear absorption seen in Ag‐Co_3_O_4_ nanostructures is due to plasmon‐assisted ESA and charge transfer between Ag NPs and Co_3_O_4_. When exposed to a 532 nm laser, LSPR in Ag NPs creates a strong electromagnetic field and generates energetic, or hot, electrons. These electrons can move across the Ag/Co_3_O_4_ interface into the semiconductor, increasing the number of excited carriers and allowing more photons to be absorbed through ESA. Simultaneously, the interface helps separate electron–hole pairs, reduces recombination, and maintains more carriers in the excited state, which improves the RSA response and optical limiting. Similar processes, in which hot electrons are injected and carriers relax rapidly, have been observed in other metal–oxide systems, such as Ag/TiO_2_ and Au/TiO_2_, using femtosecond transient absorption and pump–probe spectroscopy [70, 71, 72]. For example, Zhang et al. used femtosecond transient absorption spectroscopy to study Ag/TiO_2_ and found that hot electrons transfer from Ag NPs to TiO_2_ in about 150 femtoseconds, with charge recombination occurring within the next few picoseconds [73]. Jiang et al. also studied Co_3_O_4_ using femtosecond core‐level transient absorption spectroscopy and found that carrier relaxation occurs within a few hundred femtoseconds, indicating that short‐lived excited states play a role in charge transfer and nonlinear optical effects [74]. Although these studies were conducted on similar systems rather than directly on Ag–Co_3_O_4_, they strongly support the idea of plasmon‐assisted charge transfer and ESA. In this study, we used Z‐scan measurements, which probe the overall nonlinear optical response and provide indirect evidence of these fast carrier dynamics. Therefore, the plasmon‐assisted ESA mechanism we propose is based on our results and previous ultrafast spectroscopy studies, but it is not direct proof of hot‐electron injection or carrier lifetimes in Ag‐Co_3_O_4_. Direct evidence would require ultrafast spectroscopic methods, such as femtosecond transient absorption or time‐resolved photoluminescence, which are not part of this study but will be explored in future studies.

## 4 Conclusions

AgCo BNPs with an Ag‐rich alloy core and surface Co_3_O_4_ shell exhibited a strong third‐order nonlinear optical response under both CW and nanosecond excitation. The composite architecture couples LSPR‐driven field amplification with hot‐carrier injection into mixed‐valence Co^2+^/Co^3+^ intermediate states, establishing a sequential ESA mechanism that drives reverse saturable absorption and optical limiting. The measured β_eff_ values surpass those of several reported bimetallic systems, and χ^(3)^ = 4.12 × 10^−11^ esu places the material among high‐performing plasmonic nanocomposites, with an optical limiting onset as low as 355 W/cm^2^. These results establish that engineering a metal–semiconductor surface junction within BNPs is an effective strategy for amplifying third‐order NLO performance. Looking ahead, systematic tuning of the Co_3_O_4_ shell thickness and Ag:Co stoichiometry could further optimize the interfacial charge transfer efficiency, whereas ultrafast transient absorption studies would directly resolve hot‐carrier injection dynamics. The inherent magnetic properties of cobalt also open pathways toward magneto‐optical switching and plasmon‐driven photocatalytic applications, positioning AgCo BNPs as a versatile multifunctional platform for next‐generation photonic devices.

## Author Contributions

**T. K. Nanditha:** conceptualization, investigation, methodology, validation, formal analysis, data curation, writing – review and editing, writing – original draft, visualization. **Shreepooja Bhat:** investigation, writing – review and editing, methodology, visualization. **K. B. Manjunatha:** conceptualization, writing – original draft, writing – review and editing, methodology, validation, supervision, data curation, formal analysis, visualization. **Dileep Ramakrishna:** investigation, methodology, supervision, writing – original draft, writing – review and editing. **K. G. Raghavendra:** investigation, supervision, data curation, methodology, writing – review and editing. **S. C. Gurumurthy:** conceptualization, methodology, formal analysis, data curation, investigation, supervision, writing – original draft, writing – review and editing.

## Conflicts of Interest

The authors declare no conflicts of interest.

## Supporting information


Supporting Information S1

## Data Availability

The data that support the findings of this study are available from the corresponding author upon reasonable request.
