# Supplementary material for: Third‐Order Nonlinear Optical Response and Optical Limiting in Silver‐Cobalt Bimetallic Nanoparticles
Source: Nanophotonics. 2026 Aug 20;15(16):e70255. doi: 10.1002/nap2.70255 (PMC13672146; doi:10.1002/nap2.70255)
Supplement: Supplementary file 1 [file NAP2-15-e70255-s001.docx]

**Supporting Information**

**Third-Order Nonlinear Optical Response and Optical Limiting in Silver-Cobalt Bimetallic Nanoparticles**

*Nanditha Thayyath Kizhakkeveettil^1^, Shreepooja Bhat^1^, K. B. Manjunatha^2^, Dileep Ramakrishna^3^, Raghavendra Karkala Gururaj ^1^, Gurumurthy Sangam Chandrasekhar ^1*^*

*^1^Manipal Institute of Technology, Manipal Academy of Higher Education, Manipal, India.*

*^2^Nitte (Deemed to be University), NMAM Institute of Technology (NMAMIT), Department of Physics, Nitte, 574110, Karnataka, India*

*^3^Department of Chemistry, RNS Institute of Technology, Channasandra, Bengaluru, 560098, India*

**Corresponding Author:** [gurumurthy.sc@manipal.edu](mailto:gurumurthy.sc@manipal.edu)

**Figure S1.** Particle size distribution of Ag-Co BNPs

**Figure S2.** Rietveld refinement profiles of (a) pure Ag nanoparticles (Ag NPs) and (b) AgCo bimetallic nanoparticles (AgCo BNPs) obtained using GSAS-II.

**Table S1.** Rietveld refinement parameters and reliability factors for pure Ag NPs and Ag-Co BNPs obtained using GSAS-II.

| **Sample** | **Space group** | **Lattice parameter, a (Å)** | **R_wp_ (%)** | **R^2^_F_ (%)** | **R_F_ (%)** |
| --- | --- | --- | --- | --- | --- |
| Ag NPs | Fm-3m | 4.0880 | 6.542 | 30.159 | 16.570 |
| AgCo BNPs | Fm-3m | 4.08712 | 4.506 | 11.850 | 5.937 |
